# Supplementary material for: Insights into the Composition and Antibacterial Activity of Amomum tsao-ko Essential Oils from Different Regions Based on GC-MS and GC-IMS
Source: Foods. 2022 May 12;11(10):1402. doi: 10.3390/foods11101402 (PMC9141665; doi:10.3390/foods11101402)
Supplement: Supplementary file 1 [file foods-11-01402-s001.zip › polymers-1682403-supplementary.pdf]

Supplementary Materials

Core/Shell Pigments with Polyaniline Shell: Optical and Physical–Technical Properties

Tatyana A. Pugacheva [1], Georgiy V. Malkov [1], Alexander A. Ilyin [2], Eugene A. Indeikin [2] and Vladimir G. Kurbatov [1,2,*]

[1] Institute for Problems of Chemical Physics of the Russian Academy of Sciences (IPCP RAS), Academician Semenov Avenue 1, 142432 Chernogolovka,, Russia; sinichka.71@yandex.ru (T.A.P.); gmalkov@mail.ru (G.V.M.)

[2] , Department of Chemical Technology of Organic Coatings, Yaroslavl State Technical University, Moscow Avenue 88, 150023 Yaroslavl, Russia; 471562@mail.ru (A.A.I.); eugene.indeikin@ya.ru (E.A.I.)

* Correspondence: kurbatovvg@list.ru; Tel.: +7-(49652)-2-77-10

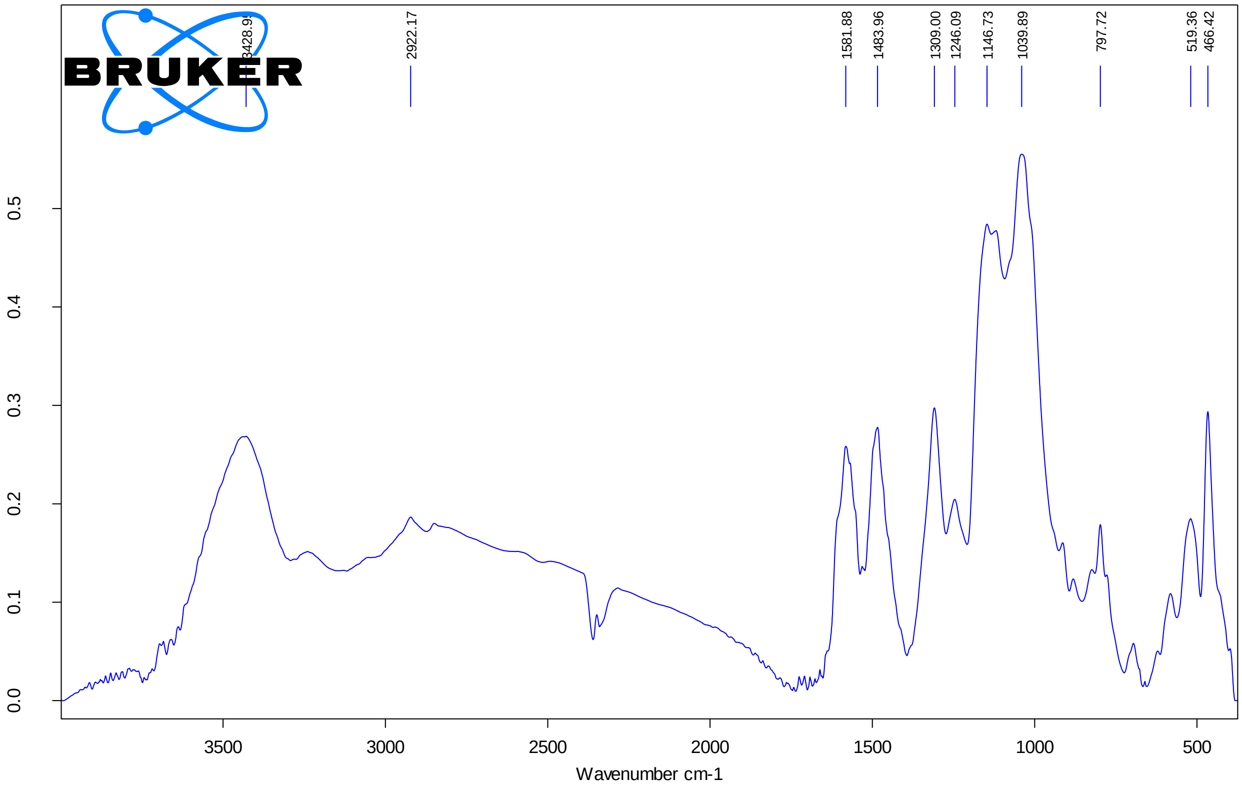

(a)

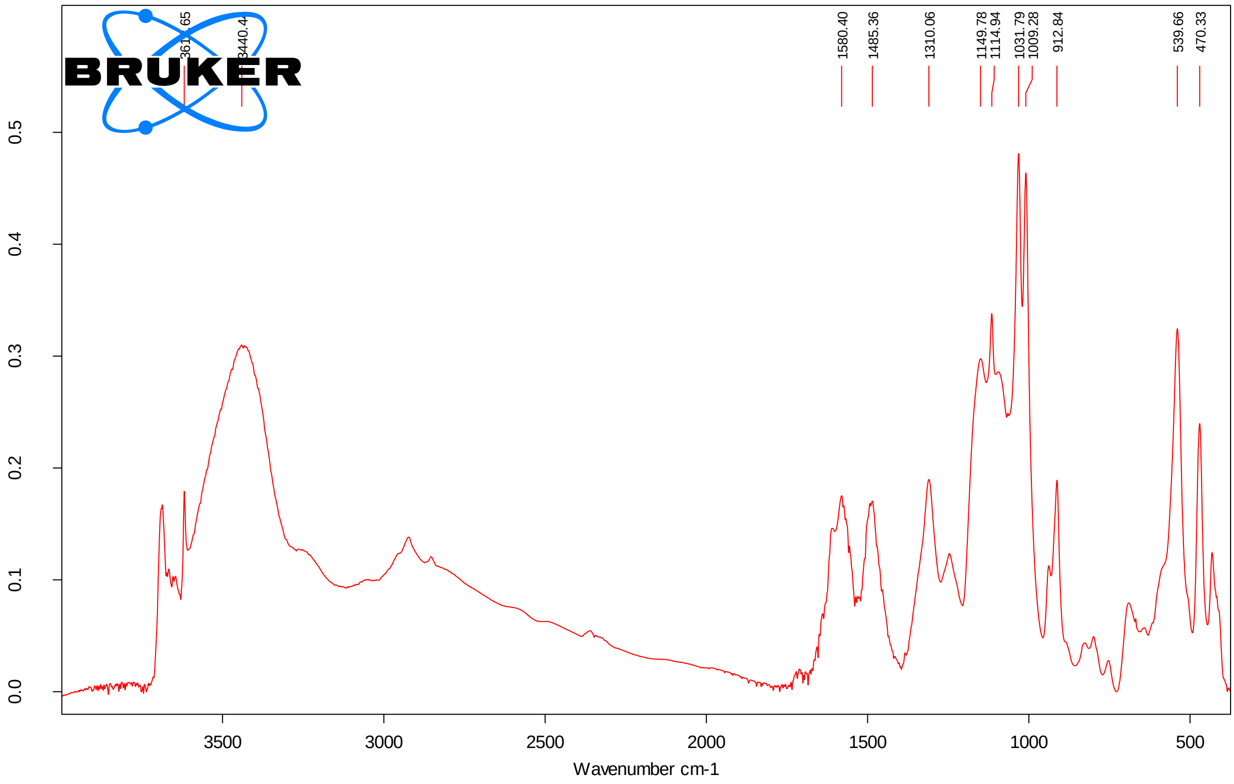
BRUKER
1580.40
1485.36
1310.06
1149.78
1114.94
1031.79
1009.28
912.84
539.66
470.33
0.0
0.1
0.2
0.3
0.4
0.5
3500
3000
2500
2000
1500
1000
500
Wavenumber cm-1

(**b**)

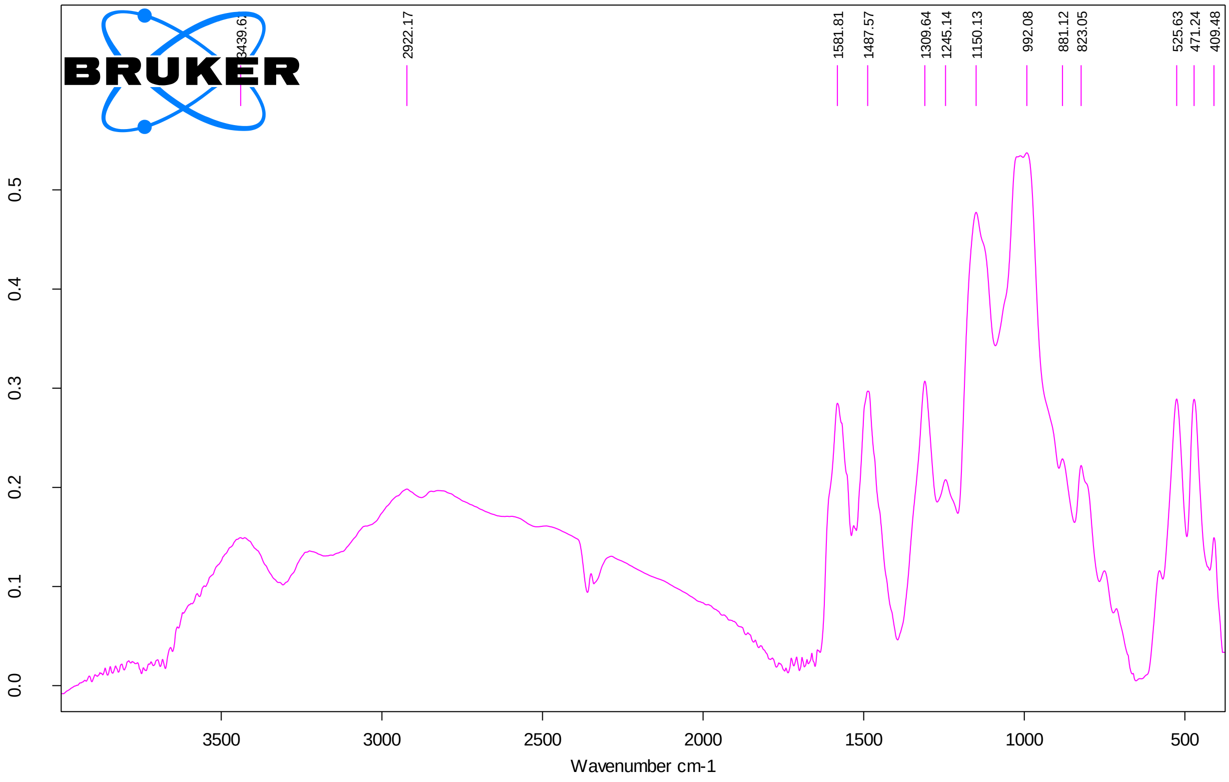
BRUKER
2922.17
1581.81
1487.57
1309.64
1245.14
1150.13
992.08
881.12
823.05
525.63
471.24
409.48
0.0
0.1
0.2
0.3
0.4
0.5
3500
3000
2500
2000
1500
1000
500
Wavenumber cm-1

(**c**)

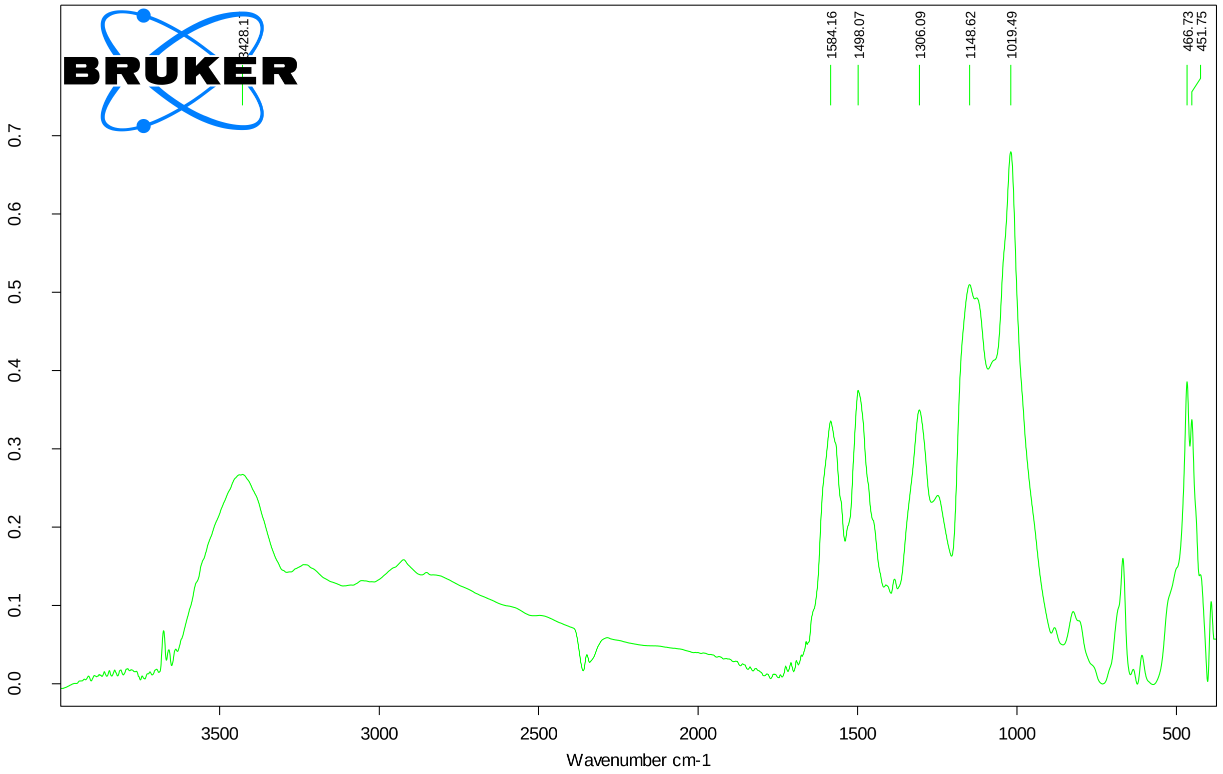

(**d**)

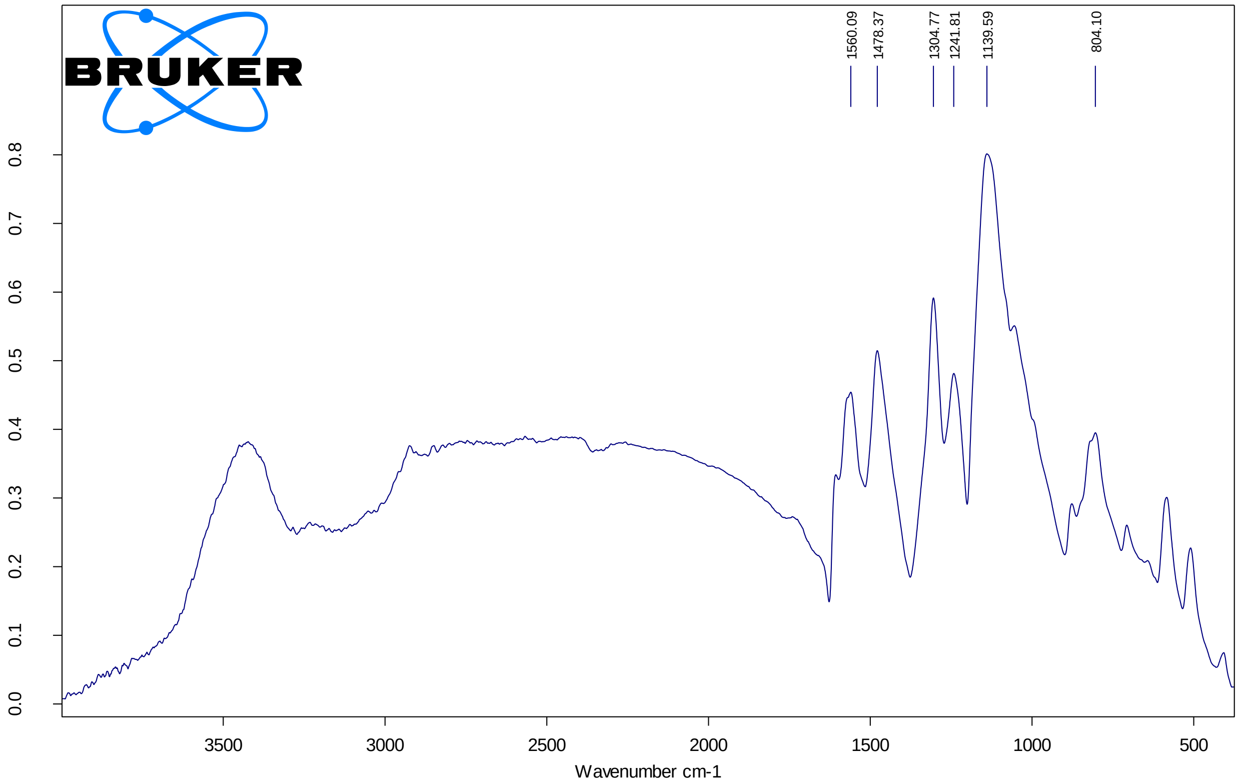

(**e**)

Figure S1. FTIR spectrum of different core/shell pigments and PANi (**a**) bentonite-based core/shell pigment; (**b**) kaolin-based core/shell pigment; (**c**) muscovite -based core/shell pigment; (**d**) talc-based core/shell pigment; (**e**) PANi doped with sulfuric acid.
